# Adverse trends in male reproductive health: we may have reached a crucial ‘tipping point’

**DOI:** 10.1111/j.1365-2605.2007.00853.x

**Published:** 2008-04

**Authors:** A-M Andersson, N Jørgensen, K M Main, J Toppari, E Rajpert-De Meyts, H Leffers, A Juul, T K Jensen, N E Skakkebæk

**Affiliations:** * University Department of Growth & ReproductionRigshospitalet, Copenhagen, Denmark; † Department of Physiology and Pediatrics, University of TurkuTurku, Finland

**Keywords:** fertility rates, male fecundity, male fertility, male reproductive health, semen quality, testicular cancer, testicular dysgenesis syndrome

## Abstract

Healthy men produce an enormous number of sperms, far more than necessary for conception. However, several studies suggest that semen samples where the concentration of sperms is below 40 mill/mL may be associated with longer time to pregnancy or even subfertility, and specimens where the concentration of sperms is below 15 mill/mL may carry a high risk of infertility. Historic data from the 1940s show that the bulk of young men at that time had sperm counts far above 40 mill/mL with averages higher than 100 mill/mL. However, recent surveillance studies of young men from the general populations of young men in Northern Europe show that semen quality is much poorer. In Denmark approximately 40 percent of the men have now sperm counts below 40 mill/mL. A simulation assuming that average sperm count had declined from 100 mill/mL in ‘old times’ to a current level close to 40 mill/mL indicated that the first decline in average sperm number of 20–40 mill/mL might not have had much effect on pregnancy rates, as the majority of men would still have had counts far above the threshold value. However, due to the assumed decline in semen quality, the sperm counts of the majority of 20 year old European men are now so low that we may be close to the crucial tipping point of 40 mill/mL spermatozoa. Consequently, we must face the possibility of more infertile couples and lower fertility rates in the future.

Adverse trends in male reproductive health have appeared during the past half century. A world wide increase in testicular germ cell cancer has been firmly established, particularly among Caucasians (Clemmesen, 1981; Huyghe *et al.*, 2003; Bray *et al.*, 2006). In Denmark where the cancer registry was established already in 1943 there has been a four-fold increase in the incidence of this disease (Clemmesen, 1968; Jacobsen *et al.*, 2006) and a young man has now almost a 1% risk of developing a testicular tumour.

## Controversies regarding retrospective data on semen quality

Parallel with this development in testis cancer there has been worrying trends in semen quality. In 1992 we published a meta analysis on published data from all parts of the world (Carlsen *et al.*, 1992) showing a decline in sperm counts, which had also been suggested by previous authors, although generally denied by prominent andrologists. The Carlsen paper received considerable attention, but in contrast to the reports on rising incidences in testis cancer, it has also caused controversy (see review by Jouannet *et al.*, 2001).

**Figure 1:**
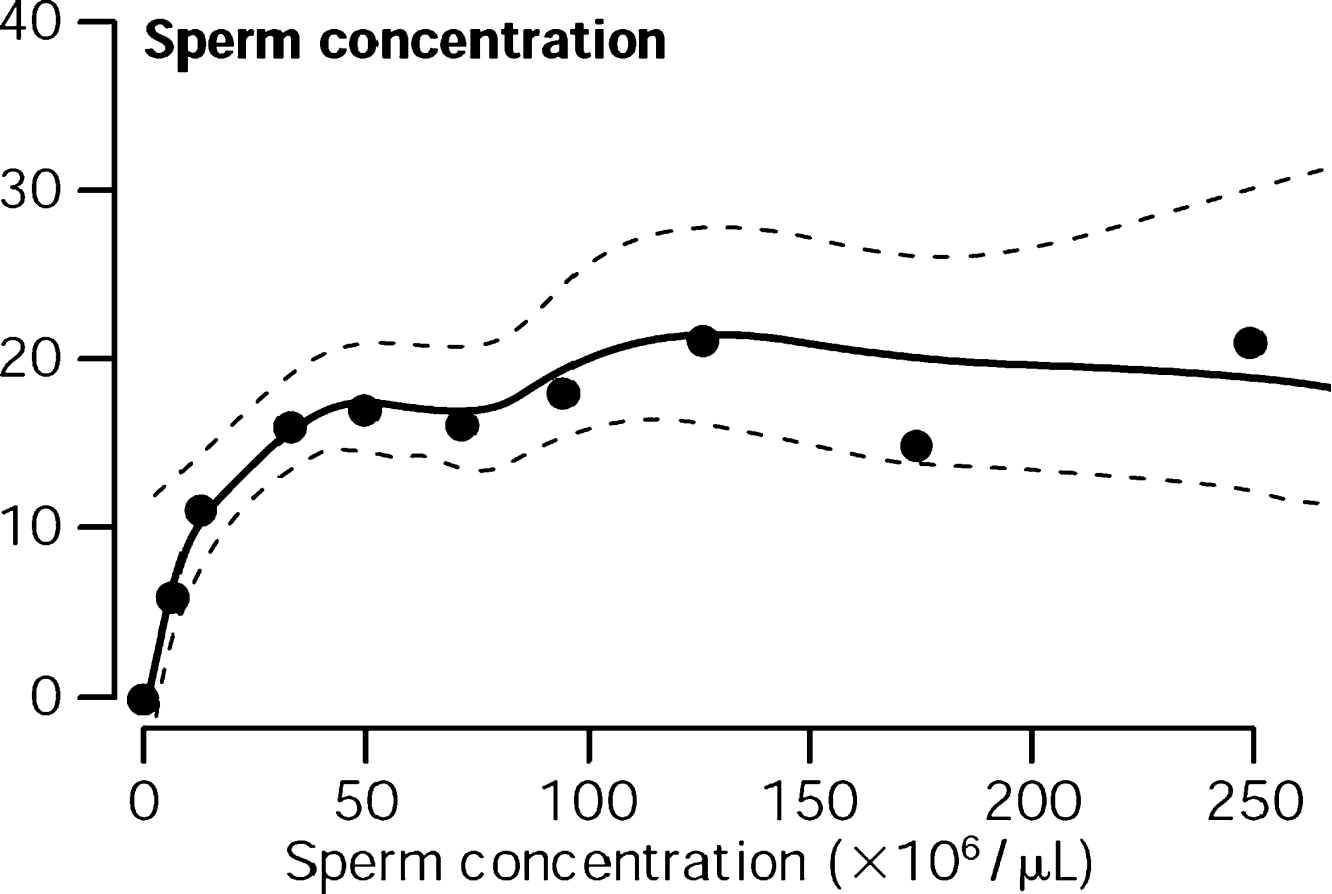
From Bonde *et al.* (1998) Lancet: 430 couples with no previous reproductive experience, aged 20–35 years participated in a study on association between semen quality and the probability of conception in a single menstrual cycle. The couples discontinued use of contraception, and were followed up for six menstrual cycles or until a pregnancy was verified within this period. Each man was asked to provide a semen sample at enrolment. Women kept a daily record of vaginal bleeding and sexual activity. The association between semen quality and likelihood of pregnancy was assessed by logistic regression, adjusted for sexual activity and female factors associated with low fertility. There were 256 (59.5%) pregnancies among the 430 couples: 165 (65.0%) among those with a sperm concentration of 40 mill/mL or more, and 84 (51.2%) among those with lower sperm concentrations. The probability of conception increased with increasing sperm concentration up to 40 mill/mL, but any higher sperm density was not associated with additional likelihood of pregnancy.

It is an interesting question, why the reports on trends in testis cancer were more or less taken ad notam, whereas the papers on adverse trends in semen quality became controversial (Jouannet *et al.*, 2001), as both trends seem to reflect the same patho-physiological problem: a deteriorating male reproductive health. However, cancer registries are often very complete and reliable, at least in Northern European countries (Adami *et al.*, 1994). In contrast, historic data on semen quality collected for various purposes are obviously less homogeneous mainly due to selection bias and differences in methods causing noise in the analyses (Jouannet *et al.*, 2001). Furthermore, sperm concentration may be drastically reduced without effects on time to pregnancy (Bonde *et al.*, 1998) and thereby fertility rates (Fig. 1). Therefore the apparent decrease in sperm counts and other semen quality variables might neither have been transmitted into a noticeable increase in time to pregnancy nor to an increase in infertility rates.

## Recent prospective studies on trends in male reproductive health

Importantly, retrospective studies on trends in semen quality have recently been followed up by controlled, prospective investigations on several male reproductive health variables in populations, including pathophysiological aspects such as cryptorchidism (Boisen *et al.*, 2004), hypospadias (Virtanen *et al.*, 2001; Boisen *et al.*, 2005) as well as physiological levels of male reproductive hormones (Andersson *et al.*, 2007; Travison *et al.*, 2007) and semen quality among normal men (Andersen *et al.*, 2000; Jørgensen *et al.*, 2001, 2002, 2006; Punab *et al.*, 2002; Richthoff *et al.*, 2002) (Fig. 1). A clear pattern is emerging that semen quality of young men in Northern Europe is generally quite poor (Jørgensen *et al.*, 2008; Paasch *et al.*, 2008) and the incidence of hypospadias and cryptorchidism may be rising, at least in Denmark, where careful studies have been performed (Boisen *et al.*, 2004, 2005). We have been surprised that we year after year have confirmed the presence of extraordinarily poor semen quality among otherwise healthy young men from the general population (Jørgensen *et al.*, 2006). According to Bonde *et al.* (1998) a semen sample should ideally contain more than 40 mill/mL in order to be optimally fertile. Other recent publications are in line with this estimate. American and European studies suggested 48 mill/mL and 55 mill/mL, respectively as lowest values of the normal range for sperm counts (Guzick *et al.*, 2001; Slama *et al.*, 2002).

**Figure 2:**
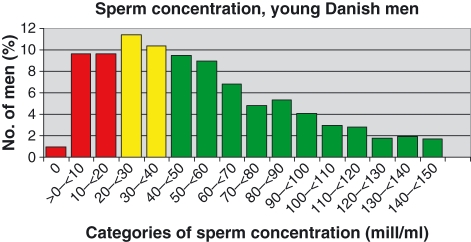
Data from a recent surveillance project on semen quality including 3517 young men from the general population in Copenhagen (Jørgensen *et al.*, 2006). The project was carried out during the years 1996–2005. The bars show the percentage of men according to categories of sperm concentration. The median sperm count of the population was 46 mill/mL during this period. Note that 42% of men had sperm counts below 40 mill/mL and therefore may belong to an at risk group of subfertility (Bonde *et al.*, 1998). 20% were even below the WHO demarcation line of for subfertility (20 mill/mL).

Unfortunately we have few normative data for semen quality from ‘old days’. However, there are some and it is interesting that our great nestors in andrology, Dr. Richard Hammen (Hammen, 1944) in Copenhagen and Dr. John McLeod (MacLeod, 1946) in New York in the 1940s suggested that a normal semen sample should at least contain 60 mill/mL sperms or more. McLeod wrote ‘I think that if we are to select a count level to represent the demarcation line between “poor and fair” fertility that of 60 mill/cc would be a wise choice’. He seemed to base his opinion on an investigation on 100 medical students, of which more than 65 had a sperm count that exceeded 100 mill/mL (MacLeod & Heim, 1945). For comparison we have consistently found that the median young man from the general population of Copenhagen had around 45 mill/mL (Fig. 2). Obviously we cannot control for differences in methods between studies carried out in the 1940s and current investigations. However, the haemocytometers, which were used for counting sperm cells during all years, were similar to those used for estimation of number of blood cells of which there do not appear to have been any trends since the 1940s. In addition MacLeods and Hammen were not the only researchers to report high sperm numbers; another excellent andrologist Hotchkiss in 1941 wrote a paper on semen quality among 200 fertile males with an average sperm number of 121.6 mill/mL, far higher that those of recently studied partners of pregnant women, except those in Finland (Jørgensen *et al.*, 2001). Partners of pregnant women have now generally much lower values, among the lowest were Danish men with a mean sperm count of 76 mill/mL (Jørgensen *et al.*, 2001; Swan *et al.*, 2003) and men from Singapore where the average (geometric mean) was 44.7 mill/mL (Chia *et al.*, 1998).

Admittedly, McLeod in his later years changed his mind with regard to ‘normal range’ for human semen quality, and lowered the low borderline for ‘normal’ semen to 20 mill/mL (MacLeod & Gold, 1953a,b) while Dr. Alvin Paulsen (Paulsen, 1974) kept the opinion that the demarcation value between fertile and subfertile should be higher (50 mill/mL). Discussions among international andrologists about normal semen were transferred into the committees producing the WHO Guidelines for semen analysis. Since 1980 these committees have had the opinion that the cut off value for a normal semen sample should be 20 mill/mL (World Health Organization, 1980, 1987, 1992; WHO, 1999). However, our recent knowledge from the studies of Bonde *et al.* (1998), Guzick *et al.* (2001) or Slama *et al.* (2002) suggest that it may make more sense to use 40 mill/mL or even a slightly higher value to distinguish between an optimal semen sample and a specimen with reduced ability to conceive. In contrast to this, there are now plans among international andrologists to set a lower limit of normal to 10–15 mill/mL.

However, no matter where we would draw the line between normal and subfertile semen samples, partners of pregnant women most often have more than 40 mill/mL spermatozoa in their ejaculates, and apparently many more sperms than necessary for conception (Jørgensen *et al.*, 2001). This redundancy in male gametes has also been shown in animal studies; spermatogenesis can be grossly disrupted without influencing fertility rates when such males were used for breeding (Ratnasooriya & Sharpe, 1989). But a crucial question is whether we have reached a threshold where the average sperm count in our current populations of young men is so low that we will begin to see an effect on fertility rates.

## Are we at the tipping point?

**Figure 3:**
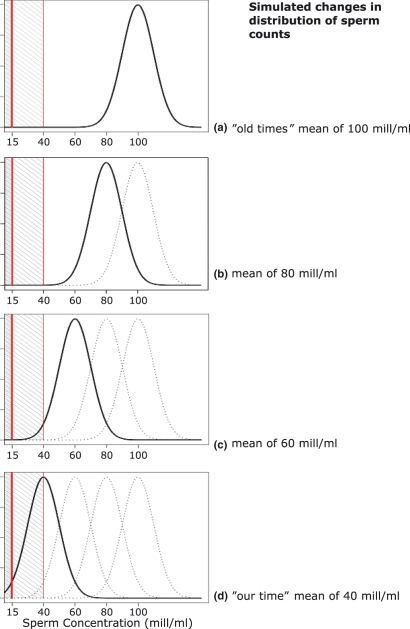
Simulation showing a gradual decrease from A to D in average sperm counts of a population from 100 mill/mL (Similar to data from studies of the 1940s) to 40 mill/mL (close to the current situation). For simplicity the simulation is based on the assumption that sperm counts of men in a population are normally distributed, although distributions of sperm counts, particularly in populations with relatively low sperm counts often show a left-skewing of the distribution curves. Note the two hatched areas between the vertical lines demarcating sperm counts of <15 mill/mL (assumed high risk of subfertility) and <40 mill/mL (assumed moderate risk of subfertility). Note that a substantial decrease in sperm count does not significantly affect fecundity until average sperm count is 60 mill/mL. As the simulation (due to lack of skewing of the curves) slightly underestimates the proportion of men with the lowest sperm counts, even more men in populations with averages of 40–60 mill/mL sperms should in reality be within the hatched areas.

Figure 3 shows a simulation assuming that normal young men in the 1940s had an average sperm count of 100 mill/mL, which should be a plausible assumption considering the old papers by MacLeod (MacLeod & Heim, 1945) and Hotchkiss *et al.* (Macomber & Saunders, 1929; Hotchkiss *et al.*, 1938). A simulated reduction in average sperm count in steps of 20 mill/mL to 80 mill/mL and even further to 60 mill/mL would not result in a noticeable increase in male subfertility. On the other hand, further reduction in average sperm count of the population to 40 mill/mL (which is rather close to where we are today) would bring many men beyond the tipping point towards subfertility, using thresholds of 15 and 40 mill/mL to demarcate substantial and moderate subfertility, respectively (Bonde *et al.*, 1998; Guzick *et al.*, 2001). Unfortunately, recent studies by Jørgensen *et al.* indicate that significant proportions of our young populations of men are below this tipping point. Even many young Finnish men, who used to show high sperm counts earlier are now below the demarcation line for good semen quality according to a recent study (Jørgensen *et al.*, 2008).

## Association between decreasing male fecundity and recent low pregnancy rates?

Based on the observed trends in semen quality, Jensen *et al.* (2007b) tested the hypothesis that an observed decreasing natural pregnancy rate among native Danes may, in fact, be partly due to decreasing male fecundity. This interpretation of the data seems to be supported by a widespread and increasing use of assisted reproductive techniques during the recent years (Andersen & Erb, 2006).

As reproductive biologists we have a great challenge to explore the observed adverse trends in human reproductive health and their causes. An important hindrance for our research is not only that we for obvious reasons cannot obtain experimental human data; we are also faced with the fact that human reproduction is – compared to most animals – a slow process. Approximately 35 years pass between two generations. The time factor is important, as there is more and more evidence that a substantial number of adult male reproductive health problems may be of fetal origin (Skakkebæk *et al.*, 2001). Undescended testis and hypospadias are obviously of fetal origin, but there is now also overwhelming evidence to associate testis cancer with an adverse fetal environment. We have proposed that these conditions often may be part of a testicular dysgenesis syndrome (TDS), which may also include poor spermatogenesis and impaired Leydig cell function. According to our current thinking genetic as well as environmental factors must be taken into account. There is strong evidence from experimental animal studies and investigations of wildlife populations that fetal exposure to endocrine disrupters, including phthalates (Mylchreest *et al.*, 1998; Fisher *et al.*, 2003; Welsh *et al.*, 2007), brominated flame retardants, bisphenol A and PFAS (perfluoralcylated chemicals), can cause TDS like conditions in animals. Humans are exposed to the very same agents through modern lifestyle. The role of these agents in reproductive health problems in humans has not been fully documented, although several recent epidemiological studies have shown associations between maternal exposures to several of these endocrine disrupters and reproductive health problems of their sons of all ages, including adulthood (Jensen *et al.*, 2004, 2007a; Main *et al.*, 2004, 2006, 2007; Swan *et al.*, 2005; Damgaard *et al.*, 2006; Damgaard *et al.,* 2007).

## Perspectives

Considering the current historically low fertility rates, which in many industrialized countries are below the levels at which populations can be sustained (Lutz *et al.*, 2003), we find it very important to elucidate the possible contribution of male subfertility for the demographic changes (Skakkebæk *et al.*, 2006). From a biological point of view it is more than plausible that the well documented world wide increase in testis cancer is a ‘whistleblower’ for increasing subfertility (Skakkebæk *et al.*, 2007). Exploration of the patho-physiological mechanisms behind these trends needs much more interdisciplinary research and attention from policy makers. The rapid changes in the male reproductive variables strongly suggest that environmental factors may play a role (Toppari *et al.*, 1996). This assumption is also in accordance with the fact that there are few known causes of low sperm counts, such as Y chromosome deletions and other genetic causes (<10%) (Krausz *et al.*, 2003). In spite of many efforts the vast majority of men with poor semen quality remain without a known cause. This causes often a great deal of concern and anguish among young families. It is almost embarrassing for us andrologists that we can provide so poor answers to infertile young men as to what is behind their abnormal semen quality. If the hypothesis of fetal origin of these reproductive health problems is valid we may not see the full effect of preventive measures within the next 30 years, even if we knew how to instigate them now. We have no time to waste in our efforts to identify the causes of these health problems.
